# An interpretable machine learning‐based cerebrospinal fluid proteomics clock for predicting age reveals novel insights into brain aging

**DOI:** 10.1111/acel.14230

**Published:** 2024-06-24

**Authors:** Justin Melendez, Yun Ju Sung, Miranda Orr, Andrew Yoo, Suzanne Schindler, Carlos Cruchaga, Randall Bateman

**Affiliations:** ^1^ Tracy Family SILQ Center Washington University in St. Louis St. Louis Missouri USA; ^2^ Department of Neurology Washington University in St. Louis St. Louis Missouri USA; ^3^ Department of Psychiatry Washington University in St. Louis St. Louis Missouri USA; ^4^ Department of Biostatistics Washington University in St. Louis St. Louis Missouri USA; ^5^ Department of Internal Medicine Wake Forest School of Medicine Section of Gerontology and Geriatric Medicine Medical Center Boulevard Winston‐Salem North Carolina USA; ^6^ Department of Developmental Biology Washington University in St. Louis St. Louis Missouri USA

**Keywords:** aging, brain aging, cerebrospinal fluid, neurodegeneration, neurodegenerative diseases, proteomics

## Abstract

Machine learning can be used to create “biologic clocks” that predict age. However, organs, tissues, and biofluids may age at different rates from the organism as a whole. We sought to understand how cerebrospinal fluid (CSF) changes with age to inform the development of brain aging‐related disease mechanisms and identify potential anti‐aging therapeutic targets. Several epigenetic clocks exist based on plasma and neuronal tissues; however, plasma may not reflect brain aging specifically and tissue‐based clocks require samples that are difficult to obtain from living participants. To address these problems, we developed a machine learning clock that uses CSF proteomics to predict the chronological age of individuals with a 0.79 Pearson correlation and mean estimated error (MAE) of 4.30 years in our validation cohort. Additionally, we analyzed proteins highly weighted by the algorithm to gain insights into changes in CSF and uncover novel insights into brain aging. We also demonstrate a novel method to create a minimal protein clock that uses just 109 protein features from the original clock to achieve a similar accuracy (0.75 correlation, MAE 5.41). Finally, we demonstrate that our clock identifies novel proteins that are highly predictive of age in interactions with other proteins, but do not directly correlate with chronological age themselves. In conclusion, we propose that our CSF protein aging clock can identify novel proteins that influence the rate of aging of the central nervous system (CNS), in a manner that would not be identifiable by examining their individual relationships with age.

## Graphical abstract

We sought to understand how cerebrospinal fluid (CSF) changes with age to inform the development of brain aging‐related disease mechanisms and identify potential anti‐aging therapeutic targets. To do so we developed a machine learning clock that uses CSF proteomics to predict the chronological age of individuals. Additionally, we analyzed proteins highly weighted by the clock to gain insights into changes in CSF and uncover novel insights into brain aging.

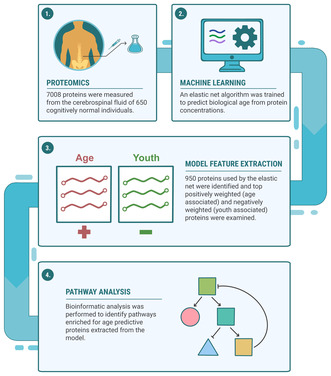

**Abbreviations**
AD: Alzheimer's disease
ADNI: Alzheimer's Disease Neuroimaging Initiative
ADRC: Knight Alzheimer Disease Research Center
ALS: Amyotrophic Lateral Sclerosis
Aβ: amyloid beta
Aβ+: amyloid beta plaque positive
Aβ‐: amyloid beta plaque negative
CDR: Clinical dementia rating
CNS: central nervous system
CSF: cerebrospinal fluid
MAE: mean estimated error
PD: Parkinson's disease

## 1 INTRODUCTION

As the brain ages, humans experience decreased mental processing speed (Manard et al., 2014) and an increased likelihood of dementia and neurodegenerative disease (Boyle et al., 2013). The composition of cerebrospinal fluid (CSF) also changes with age: total protein turnover slows (Chen et al., 2012), the blood–brain barrier becomes more permeable to molecules from plasma (Knox et al., 2022), and expression in the choroid plexus is altered (Tahira et al., 2021). These changes to proteins in the extracellular environment of the central nervous system (CNS) may contribute to proteostatic dysregulation in brain tissue, accumulation of toxic molecules, and impairment of mechanisms preventing neurodegeneration (Marques et al., 2013). Additionally, the prevalence of age associated diseases such as late onset Alzheimer's disease (AD) changes over time, with the incidence of AD doubling every 5 years after age 65 (Qiu et al., 2009).

Understanding the proteomic changes that occur during aging in biological fluids may provide new therapeutics for neurodegenerative disorders and brain health‐span extension. For example, administering CSF (Iram et al., 2022) from young animals into older animals has beneficial therapeutic effects on memory, health, and cognition. However, more work needs to be done to understand the mechanistic changes underlying these benefits and effects on aging. In order to determine if interventions reverse or delay aging, objective metrics, that is, biomarkers, that can measure the age of an organism or tissue are needed.

To this end, there has been great interest in developing aging “clocks” that can predict the age of an organism from biomarkers. Deviations in these clocks from expected chronological age can be used to determine if interventions are effective at modulating aging. The majority of these clocks have been based on changes in epigenetic methylation marks on DNA in plasma or tissues (Horvath, 2013), (Zhang et al., 2019), (Johnson et al., 2020). Brain cell and brain tissue‐specific clocks have also been developed that show excellent correlations with chronological age (Buckley et al., 2023; Horvath, 2013) including epigenetic plasma clocks that have been optimized for brain aging (Cole, 2020).

However, several major challenges remain that limit the utility of these clocks to understand brain aging and identify testable protein targets for therapeutic intervention. While many robust plasma‐based epigenetic aging clocks have been developed, they have proven insufficient in reflecting neuropathologies associated with brain aging (Fransquet et al., 2021). Brain‐ and tissue‐specific clocks provide a more direct measurement of brain aging; however, these clocks cannot typically be used in living patients due to lack of access to samples. While methylation‐based surrogates that correlate with protein concentration have been developed and proven valuable in diagnosing disease (Fu et al., 2021), greater predictive power might be obtained by measuring protein concentration directly. Thus, there is need for a proteomics‐based clock that is both reflective of brain aging and acceptable for use in living individuals.

Recently, several novel minimally invasive clocks have arisen that do not rely on epigenetics. Notably, MRI clocks which use imaging of brain volumes and other metrics to predict age (Cole, 2020), and a proteomics based blood clock which uses blood circulating proteins to infer the ages of organs throughout the body (Oh et al., 2023). We believe these are large steps forward as imaging‐based clocks can be used in live subjects and organ‐age inference clocks provide information about multiple regions of the body from easily obtainable plasma. However, neither of these clocks directly measures the proteomic changes that occur in the aging brain environment. Proteins are the machinery of life; they are the direct drivers of the reactions that make up biology and the end point for biological functionality. This makes their impact on aging directly interpretable and hypotheses generated from their study testable. Thus, we believe there is great need for a CSF proteomics clock that can measure CSF aging, provide direct insight into the pathways and proteins that change during age, and uncover new targets for brain aging and neurodegeneration interventions.

**FIGURE 1:**
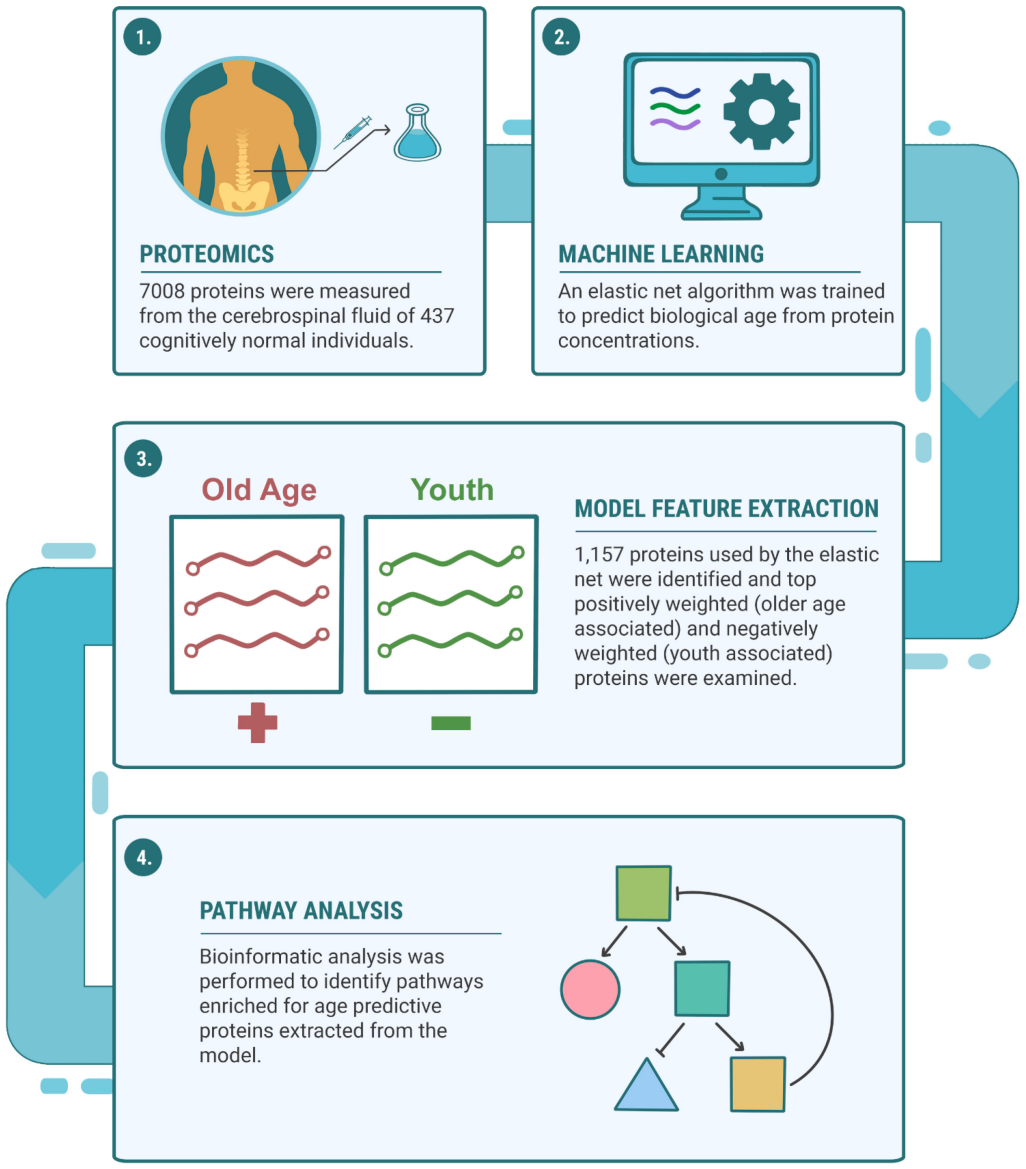
A graphical overview depicting development of the CSF aging clock, model feature extraction, and bioinformatics. (1) CSF from 437 cognitively normal, amyloid beta negative individuals were measured using the SomaLogic system to quantify 7008 proteins. (2) An elastic net was trained on the proteomics profiles to develop a machine learning algorithm that predicts biological CSF age. (3) Proteins used to predict age were extracted from the model, ranked and categorized into older associated and younger associated groups. (4) Pathway enrichment was performed via the bioinformatics tool Metascape to look for pathways enriched in model proteins in the Reactome pathway knowledgebase.

In this study, we present a machine learning‐based aging clock that uses human CSF proteomics data as an input to predict the chronological age of the CNS. As CSF is readily available via lumbar punctures, it provides an opportunity to approximate brain aging in living persons. CSF is routinely banked by many cohorts studying age‐related brain diseases, such as Parkinson's disease (PD) and AD making it an accessible biofluid to understand the impact of diseases and treatments on brain aging. While taking CSF is a more invasive process than blood draws, it is drawn regularly in some parts of Europe and it's use has significantly increased in memory and neurology clinics to allow for accurate diagnosis of Alzheimer's disease. We selected CSF proteomic measures from cognitively normal, non‐AD controls to investigate age‐related changes in CSF. We then implemented a machine‐learning algorithm to build a CSF proteomics biologic clock to identify specific age‐related proteins, allowing a physiologic interpretation of the aging proteome. Here we demonstrate that our clock has high concordance with chronological age, identifies an interpretable set of proteins and pathways used to estimate biological CNS age, and provides insights into the biological processes of CNS aging. We hypothesize that negatively weighted proteins used by the model to predict age will be associated with protective effects on age‐associated brain pathologies and decline (i.e., youth‐associated mechanisms), and that loss of these proteins will be associated with harmful aging phenotypes. Likewise, we hypothesize many proteins with positive weights will be associated with detrimental CNS aging‐associated phenotypes or compensatory responses to aging. Highly weighted proteins may identify potential targets or pathways for aging interventions. To explore these hypotheses, we analyzed the top highly positively and highly negatively weighted proteins identified by our model and found age‐associated effects in the literature. We also explored age predictive changes on a systems level by examining pathways that are highly enriched for proteins used by our model to predict age (Figure 1). In addition to our full model, we also showcase a more compact minimal model, that uses an order of magnitude fewer proteins, with similar performance and a method to generate such compact models. Finally, we tested our clock on a second validation cohort and a group of cognitively normal but amyloid beta plaque positive (Aβ+) individuals to see if this presymptomatic AD group demonstrated accelerated biological aging in the CNS.

## 2 MATERIALS AND METHODS

### 2.1 Cohorts

Demographic, diagnostic, and CSF proteomics data for this study was obtained via a data request to the Knight Alzheimer Disease Research Center (ADRC). Proteomics data was generated by the NeuroGenomics and Informatics (NGI) Center at Washington University and protein concentrations were measured via the SomaLogic proteomics platform. The original cohort consisted of 857 participants with varying ranges of Clincial Dementia Rating (CDR) and AD pathology. To remove bias from age related disease states, such as AD and other forms of dementia, we narrowed our cohort down to 437 cognitively normal participants with CDR = 0, and Aβ42/40 ratios more than 0.0673 as the cutoff for amyloid positivity (Barthélemy et al., 2023). Aβ42/40 ratios were measured via Lumipulse. Participant age ranged from 43 to 91 years old with a median age of 69. Regarding sex at birth, 249 of the participants were female and 188 were male. Participant years of education ranged from a minimum of 9 years to a maximum of 24 years with a median of 16 years. Race of the cohort was as follows: 382 White (non‐Hispanic), 52 Black, two people of mixed race and one of Asian descent. A second validation cohort was obtained from the Alzheimer's Disease Neuroimaging Initiative (ADNI). The full cohort included 735 individuals for which there was CSF proteomics data available. The subcohort of cognitively normal amyloid negative individuals consisted of 44 individuals with CDRs of 0. CSF measurement on the Elecsys platform of pTau/Aß42 < =0.025 was used to determine amyloid positive status. This threshold was determined by ROC analysis that gave the best sensitivity and specificity with PET centiloid scores used for amyloid beta status classification.

### 2.2 Machine learning and elastic net model

A custom python program was built to train an elastic net‐based machine learning algorithm to predict participant chronological age based on 7008 protein measurements (features) in human CSF. The elastic net algorithm was implemented via the scikit‐learn machine learning library. Missing data points were imputed using a k nearest neighbor‐based approach with *K* = 2. Prior to training the data was scaled from 0 to 1 using the min‐max feature scaler of scikit‐learn, which gave the best performance of the data transformation approaches tried. Additional scaling approaches explored included log transforming the data, the scikit‐learn standard scaler and the scikit‐learn power transformer (which also normalizes unit‐variance). To generate the model, the data were randomly split into two sets: a “training set” consisting of 349 individuals (80% of the dataset) and a “test set” (20% of the dataset) consisting of 88 individuals held aside to validate the model. The training set was then split again into two groups consisting of 279 individuals (80% of the training set) and 70 individuals (20% of the training set). This first set, the “sub training set,” was used to train the elastic net and find the optimal tuning parameters (L1 and Alpha), while the second set, the “sub validation set,” was used as validation data for this stage of the training. The tuning parameters of the final model were an Alpha of 0.0012 and an L1 of 0.58. After optimal tuning parameters and feature weights were determined, a third order polynomial, the “transformation polynomial,” was fit on the age predicted by the model on the sub validation set and the real chronological age of the participants. The polynomial used in the final validation of the model took the form: y = 68.5 + 16.7·x – 4.0·x^2^–0.8·x^3^, where x is the original prediction made by the model and y is the adjusted prediction. Subsequently a linear transformation was trained to shift the data such that a linear regression fit to the data had an x and y intercept of approximately zero. The linear shift took the form y = ((60.7 – x)/0.82) + 60.7. Weights for both the polynomial and linear transformations were trained on the training data only and at no point had access to the final validation data used in figures. When making final predictions for the validation group, the model was given access only to the 7008 SomaLogic measured protein concentrations and had no other information about the validation group participants.

### 2.3 Feature extraction

Upon training of the final model each protein feature was given a weight based on how much it was used in the final model to predict chronological age. Features with a weight of zero were not used at all by the model. Features with a positive weight contributed to increased estimations of age, whereas features with negative weights contributed to decreased estimations of age.

### 2.4 Pathway enrichment

The bioinformatic tool Metascape (Zhou et al., 2019) (https://metascape.org) was used to find pathways in the Reactome knowledge base (Gillespie et al., 2022) that were enriched for features used by the final model to predict age. Metascape is a tool designed to analyze and interpret OMICs‐based data, and Reactome is a manually curated database that provides molecular details across a broad range of physiological and pathological biological processes in humans. Settings for Metascape included a minimum overlap of 3, *p* value cutoff of 0.01 and a minimum enrichment score of 1.5. A total of 1157 model weighted human protein features were used in the enrichment search and all 7008 possible protein features were used as a background.

### 2.5 Statistical analysis

A Pearson correlation between model predicted ages and chronological ages was used as a measure of model accuracy. Models were trained to maximize this correlation during the training phase. *R* ^2^ values were generated by fitting a linear regression between model predicted ages and chronological ages. Correlation with chronological age was generated via spearman correlation across the entire cognitively normal, Aβ plaque negative (Aβ‐) cohort. A one tailed *t*‐test was used to test the hypothesis that Aβ plaque positive (Aβ+) status in cognitively normal individuals would accelerate biological aging.

### 2.6 Iterative re‐weighting and down‐sampling

An elastic net machine learning algorithm was used to generate our full 1157 protein feature CSF aging clock as described above. These protein features were then ranked by absolute value of weights given by the full model. The bottom five least weighted protein features were then removed from the dataset, and the model was retrained on the remaining proteins to generate a new ranking. This process was then repeated 230 times until only four proteins remained from the original 1157 proteins. Pearson correlation values were then calculated and plotted versus proteins used by each model to generate Figure 4a.

## 3 RESULTS

### 3.1 Machine learning model

**FIGURE 2:**
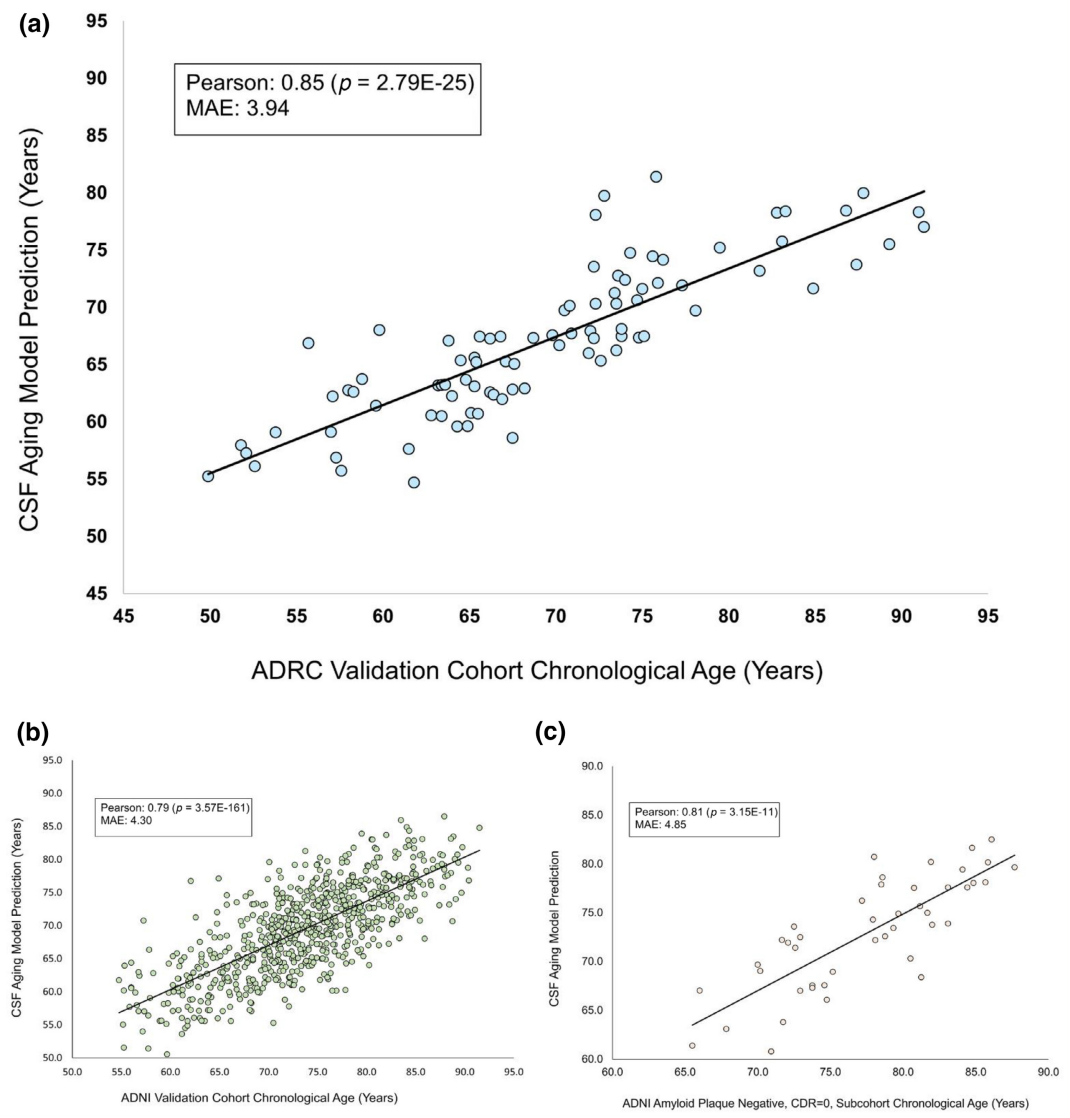
Age predicted via CSF proteomics elastic net model versus chronological age. An elastic net was trained to predict chronological age from SomaLogic CSF proteomics profiles, each containing measurements of 7008 different proteins. (a) The model was trained on 349 cognitively normal, amyloid beta negative, participants from the Knight ADRC, ages 43–91 with a median age of 69. The trained model is shown predicting the ages of an additional 88 cognitively normal participants not included in the training data. The model made use of 1157 protein features to generate predicted CSF age and shows a 0.85 Pearson correlation with chronological age (*p* = 2.79E‐25) and a mean average error of 3.94 years. (b) The trained model was then validated using CSF proteomics from an additional cohort, the Alzheimer's Disease Neuroimaging Initiative (ADNI) consisting of a new set of 735 individuals. The model validated on this cohort with a Pearson correlation of 0.79 with chronological age (*p* = 3.57E‐161) and MAE of 4.30 years. (c) As the ADNI cohort included a range of individuals with varying clinical dementia ratings (CDR) and amyloid beta statuses a subcohort was created of 44 ADNI participants who were verified to be cognitively normal and amyloid beta negative. The model was then validated on this subcohort and had a 0.81 Pearson correlation with chronological age (*p* = 3.15E‐11) and MAE of 4.85 years.

To create a machine learning algorithm to predict chronological age from proteins in human CSF, we trained a modified elastic net on the concentrations of 7008 proteins in human CSF as measured on the Somalogic proteomics system. Of the 7008 measured CSF proteins, the model made use of 1157 proteins to predict age. Figure 2a shows the trained model's predictions of age in years (y‐axis) versus the actual chronological ages of the 88 individuals in the validation cohort (x‐axis). Predictions made by the model showed a high level of agreement with chronological age at the time of CSF draw, with a Pearson correlation of 0.85 and mean estimated error of 3.94 years. Additonally, we preformed 100‐fold cross testing of our model generation method and confirmed that our selected model was represenatative of the median Pearson correlation and MAE performance of these model distributions (Appendix S2). The trained model was then validated on the CSF proteomes of an additional 735 participants from an additional cohort, the Alzheimer's Disease Neuroimaging Initiative (ADNI). The model continued to perform well on this cohort with a Pearson correlation to chronological age of 0.79 and an MAE of 4.30 (Figure 2b). As the original model was trained on only cognitively normal, amyloid beta negative individuals, and the ADNI cohort included participants with a range of cognitive states and amyloid status, we created a subcohort of 44 individuals who we confirmed were amyloid beta negative and cognitively normal. We then validated our CSF aging model on this subcohort and obtained similar results to the full cohort, with a Pearson correlation of 0.81 and MAE of 4.85 (Figure 2c). This validation confirms the power of our model to predict chronological age from CSF proteomics.

### 3.2 Cohort‐wide protein correlation with age

**FIGURE 3:**
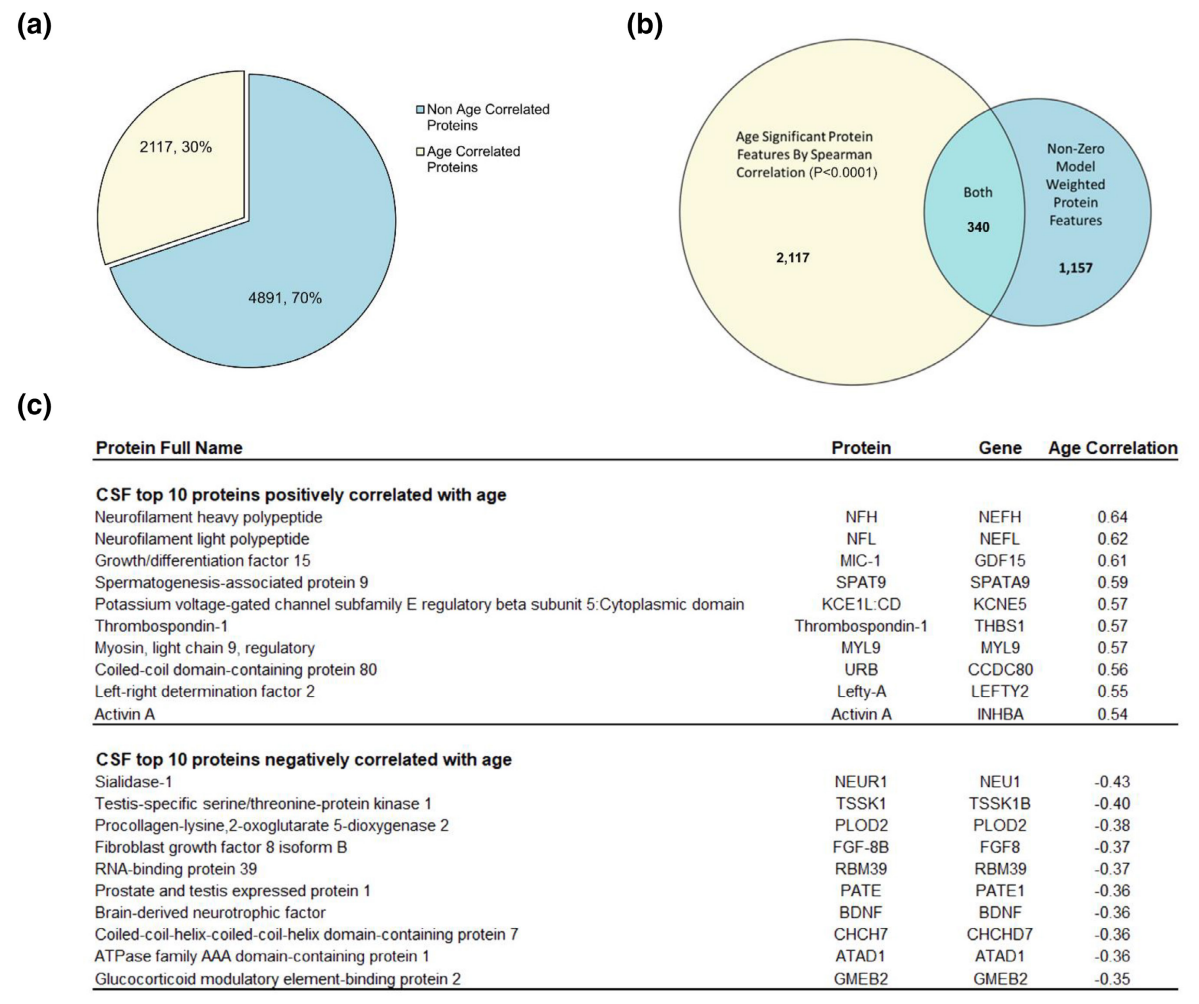
Proteins that vary significantly with age via Spearman correlation. We ran Spearman correlations with chronological age on the concentrations of all 7008 proteins measured in our cohort of CDR = 0, Aβ‐ individuals. (a) 30% (2117) of all CSF proteins measured significantly correlated with age (*p* < 0.0001) while 70% (4891) of measured proteins showed no such relationship. (b) Three hundred and forty of the 1157 proteins used in our elastic net model to predict age were found to significantly correlate with chronological age. (c) A chart showing the full names, Entrez gene IDs and correlation values of the top 10 significant positively and negatively correlated proteins with chronological age.

An additional way we sought to understand how CSF proteins in our cohort changed with age was correlation. We performed Spearman correlations with chronological age on the concentrations of all proteins in our data using *p* < 0.0001 as the threshold for significance. We observed that 2117 protein features (30%) of the 7008 possible protein features significantly correlated with age (Figure 3a). Of these, 340 age correlated proteins were used by our model (Figure 3b). Figure 3c shows the top 10 positively weighted and negatively weighted proteins that significantly correlated with age. A full list of all proteins that significantly correlated with age is available in the extended materials (Appendix S1).

### 3.3 Model feature extraction

To investigate which proteins in CSF were predictive of aging, we extracted features from our elastic net and ranked them by the model weight, an indication of how much impact each protein had on the final prediction. A positive weight indicates higher protein concentrations are predictive of older ages, whereas a negative weight indicates higher concentrations predict more youthful states. An advantage of elastic net‐based models over many other machine learning models is that they are both interpretable and excel at excluding non‐informative features, while retaining informative but redundant features by distributing their predictive weights. Of the 7008 total proteins, 1157 were used by the model to predict aging, while 5851 proteins had weights of zero indicating our model did not find them informative. Five hundred and eighty‐nine proteins had positive weights, while 568 proteins had negative weights. Table 1 summarizes the top 10 positively and negatively weighted proteins extracted from the model. A full list of all proteins and weights used by the model can be found in the extended materials (Appendix S1).

**TABLE 1:** Top proteins used to predict age by CSF proteomics model. Features used by the elastic net to predict age were extracted along with their model weights. This table shows the full names, abbreviations, Entrez gene symbols and model weights of both the top 10 positively weighted (top) and top 10 negatively weighted (bottom) proteins ordered by model weight. The table also shows each protein's spearman correlation with age across the entire dataset. Values are listed where spearman correlation with age significance met a threshold of *p* < 0.0001. NS is listed if the protein did not correlate with age significantly.

Protein full name	Protein	Gene	Age correlation
**CSF top 10 proteins positively correlated with age**			
Neurofilament heavy polypeptide	NFH	NEFH	0.64
Neurofilament light polypeptide	NFL	NEFL	0.62
Growth/differentiation factor 15	MIC‐1	GDF15	0.61
Spermatogenesis‐associated protein 9	SPAT9	SPATA9	0.59
Potassium voltage‐gated channel subfamily E regulatory beta subunit 5:Cytoplasmic domain	KCE1L:CD	KCNE5	0.57
Thrombospondin‐1	Thrombospondin‐1	THBS1	0.57
Myosin, light chain 9, regulatory	MYL9	MYL9	0.57
Coiled‐coil domain‐containing protein 80	URB	CCDC80	0.56
Left–right determination factor 2	Lefty‐A	LEFTY2	0.55
Activin A	Activin A	INHBA	0.54
**CSF top 10 proteins negatively correlated with age**			
Sialidase‐1	NEUR1	NEU1	−0.43
Testis‐specific serine/threonine‐protein kinase 1	TSSK1	TSSK1B	−0.40
Procollagen‐lysine,2‐oxoglutarate 5‐dioxygenase 2	PLOD2	PLOD2	−0.38
Fibroblast growth factor 8 isoform B	FGF‐8B	FGF8	−0.37
RNA‐binding protein 39	RBM39	RBM39	−0.37
Prostate and testis expressed protein 1	PATE	PATE1	−0.36
Brain‐derived neurotrophic factor	BDNF	BDNF	−0.36
Coiled‐coil‐helix‐coiled‐coil‐helix domain‐containing protein 7	CHCH7	CHCHD7	−0.36
ATPase family AAA domain‐containing protein 1	ATAD1	ATAD1	−0.36
Glucocorticoid modulatory element‐binding protein 2	GMEB2	GMEB2	−0.35

### 3.4 Pathways enriched for model predictive proteins

To gain further insight into the biological impact of age predictive protein changes in CSF we searched for biochemical pathways enriched for proteins used by our model. To do this we used the bioinformatics platform Metascape to search the Reactome knowledgebase for pathways significantly enriched for model weighted proteins. All 1157 positively and negatively weighted protein features were used in this analysis, and the entire set of 7008 possible proteins was used as a background. Metascape found a total of seven nonredundant pathways in which proteins in our model were significantly enriched (*p* < = 0.01). A list of enriched pathways and proteins enriched in each pathway is available in Table 2.

**TABLE 2:** Pathways enriched by model proteins. The bioinformatic tool Metascape was used to find pathways in the Reactome knowledgebase that were enriched for features used by our full model to predict age. Thousand one hundred and fifty‐seven model weighted protein features (submitted as Entrez Gene IDs) were used in the enrichment search and all 7008 possible features were used as a background. Displayed are pathways found to be significantly enriched for proteins in our model (*p* < 0.01 and enrichment score >1.5), the name of each Reactome pathway, *p* values, and the Entrez gene symbols of model proteins enriched in each pathway.

Description	*p* Value	−log10 (*p*)	Features in pathway (Entrez gene symbol)
Glycoprotein hormones	0.000008	5.08	CGA, FSHB, INHA, INHBA, INHBB, INHBC, LHB, TSHB, CES1, CPB1, ENPEP, GZMH, IGF1, P4HB, REN, PLA2G7, and GHRL
Complement cascade	0.000156	3.81	C1QC, C2, C3, C4A, C4B, C7, C8A, C8B, C8G, C9, CPN2, CR1, CR2, CFD, FCN2, CFH, CFHR2, CFP, VTN, and FCN3
Scavenging by Class A Receptors	0.000164	3.78	APOB, COL1A1, COL3A1, FTH1, FTL, HSP90B1, MARCO, COLEC12, SCARA5, SCARB1, HPX, SAA1, SPARC, and APOL1
Non‐integrin membrane‐ECM interactions	0.000953	3.02	COL1A1, COL2A1, COL3A1, COL10A1, COL11A2, DAG1, TNC, ITGA2, ITGB1, PDGFA, TGFB1, VTN, SDC3, and TRAPPC4
Mitochondrial fatty acid beta‐oxidation of unsaturated fatty acids	0.001054	2.98	ACADL, ACADM, ECI1, and DECR1
TP53 regulates transcription of death receptors and ligands	0.002879	2.54	FAS, IGFBP3, TP53, TNFRSF10D, TNFRSF10B, BCL6, BID, and TP53I3
Terminal pathway of complement	0.006540	2.18	C7, C8A, C8B, C8G, and C9

### 3.5 Generation of a minimal protein model for age prediction

**FIGURE 4:**
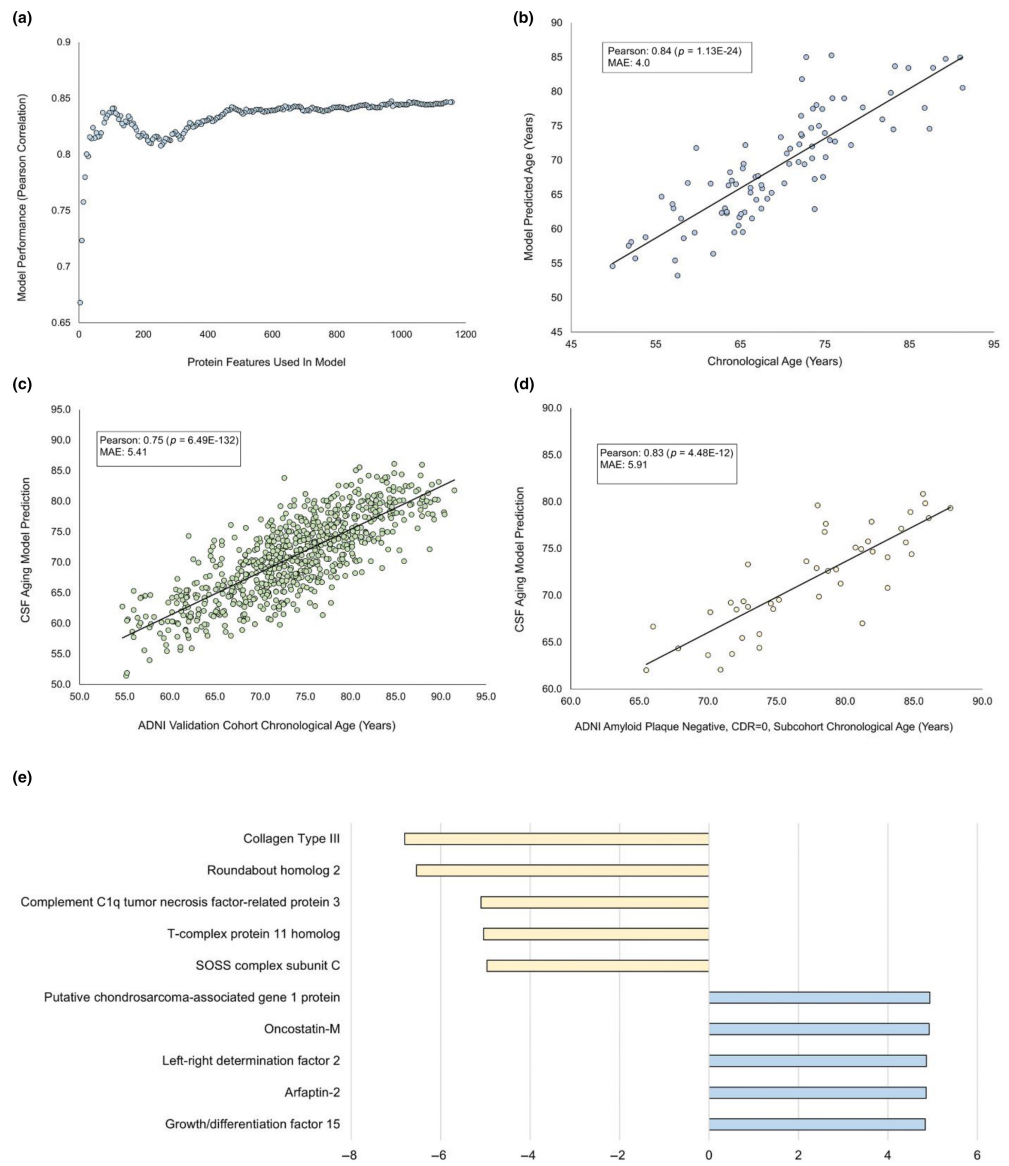
Iterative down‐sampling of proteins to create a minimal CSF aging model. An elastic net was trained on progressively fewer proteins to create a minimal model for predicting chronological age from CSF. Training began with the 1157 protein features identified in the full model. After each round of retraining, proteins were reordered by the absolute values of their new weights, and the bottom 10 proteins were eliminated. (a) The number of proteins used plotted against the Pearson correlation of the model's prediction of ages versus known chronological ages. (b) The results of a 109‐protein “minimal” model predicting age versus known chronological age on the validation cohort. This minimal model had a 0.84 Pearson correlation with chronological age of the ADRC validation group (*p* = 1.13E‐24) and an MAE of 4.0 (c) The ADRC trained 109‐protein model used to predict chronological ages of the full ADNI validation cohort. The 109‐protein model validated on this cohort with a Pearson correlation of 0.75 with chronological age (*p* = 6.49E‐132) and MAE of 5.41 years. (d) The ADRC trained 109‐protein model used to predict chronological ages of participants from the ADNI cohort who were verified to be amyloid beta negative and cognitively normal. The 109‐protein model validated on this cohort with a Pearson correlation of 0.83 with chronological age (*p* = 4.48E‐12) and MAE of 5.91 years. (e) Top 10 protein features weighted by absolute value in the 109‐protein minimal model.

In addition to the full 1157 protein model, we set out to generate a model using the fewest proteins possible while maintaining accuracy. Our goal was to create a minimal model that was more accessible to use due to requiring fewer proteomics measurements and determine the smallest set of proteins with enough information content to predict age. To do so we developed a process we called iterative feature down‐sampling, whereby we progressively retrained our elastic net using features in the previous elastic net, re‐ranking by absolute value of model weights and dropping the bottom 10. We then graphed the Pearson correlations of each model (Figure 4a). Model accuracy dropped steeply around 109 proteins, with our 109‐protein model (Pearson 0.84, *p* = 1.13E‐24) (Figure 4b) performing nearly as well as our full 1157 protein model (Pearson 0.85, *p* = 2.79E‐25) (Figure 2) on the ADRC test data. To validate the 109‐protein model on an additional cohort, the pretrained model was run on both the full ADNI cohort (Pearson 0.75, *p* = 6.49E‐132) (Figure 4c) and the subset of ADNI participants verified to be amyloid beta negative and cognitively normal (Pearson 0.83, *p* = 4.48E‐12) (Figure 4d). Top protein features were then extracted from the model (Figure 4e), and the full 109 protein features were used for pathway analysis. Notably, representatives from all pathways enriched for by the full model were present in pathways enriched for by the 109‐protein model. It is possible that these proteins contain enough age predictive information to represent the missing proteins from their respective pathways and may be of further interest biologically. A full list of all proteins in the 109‐protein model, and their training weights, is available in the Appendix S1.

### 3.6 Amyloid beta status in cognitively normal individuals does not affect the accuracy of our CSF aging clock

**FIGURE 5:**
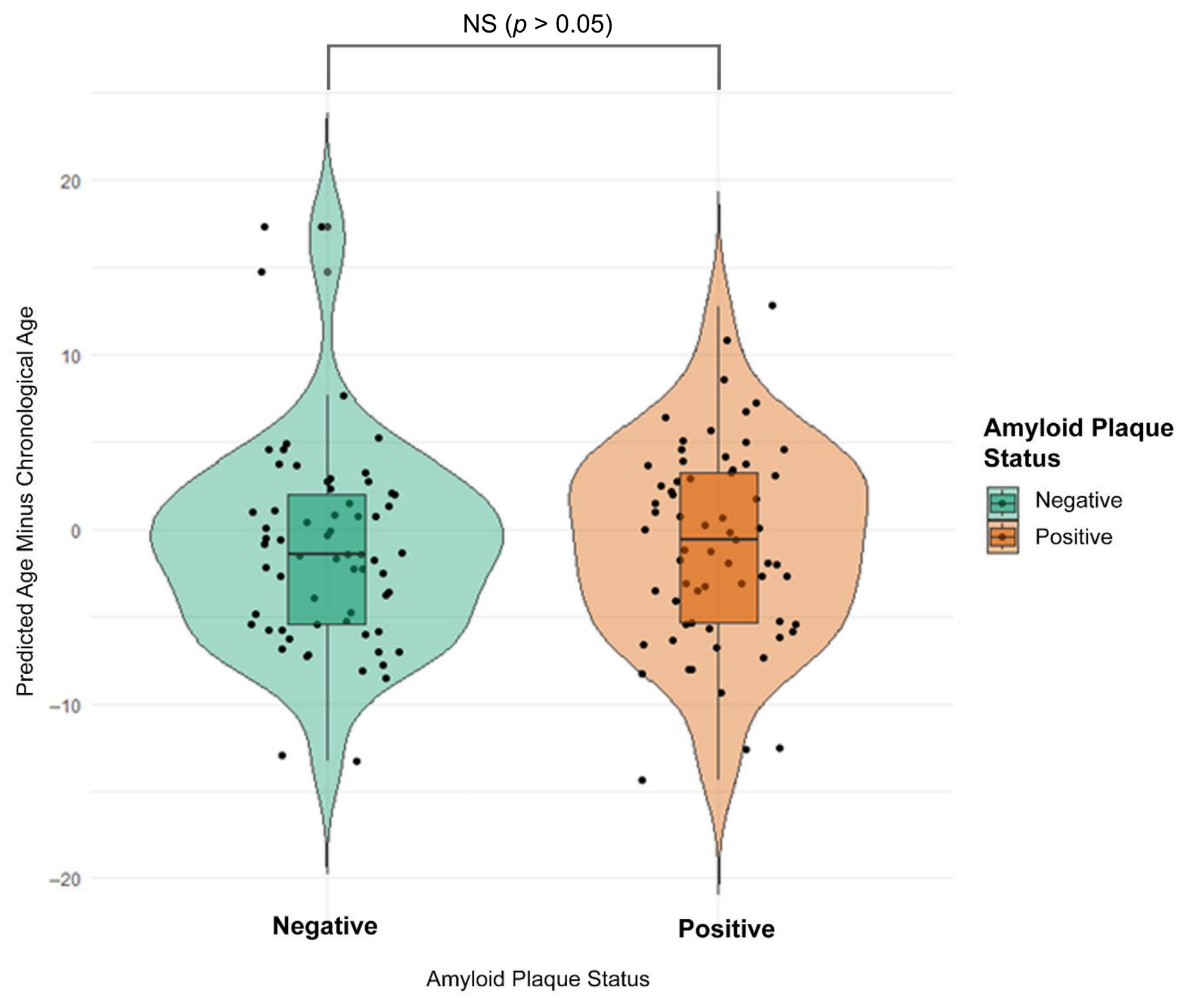
CSF aging model run on a cognitively normal (CDR = 0) group with amyloid plaques. We ran our CSF aging model on a CDR = 0 Aβ + group of 65 people previously unseen by our pretrained model and compared results to a group of 65 CDR = 0, Aβ‐, age, sex, and ethnicity‐matched participants from the validation dataset (also unseen by the model until after weights had been determined from the training set). Seen above are the differences between model predicted age and chronological age for each participant (Y axis). The Aβ‐ averaged a deviation of −1.03 years from chronological age, while the Aβ + group deviated by −0.86 years. This difference was not found to be significant by a one tailed T‐test (*P* = 0.86).

One application of our CSF proteomics aging clock was to examine if participants diagnosed with age associated diseases such as AD deviate significantly in predicted age versus healthy individuals. To do this we compared the validation cohort of our clock, who were all both cognitively normal (CDR = 0) and Aβ‐, to a new group of cognitively normal (CDR = 0) but Aβ + individuals, indicating presymptomatic AD. Each group contained a total of 65 age, race, and gender matched participants. We then applied the full 1157 protein feature clock to both groups and plotted the difference in years between clock's predicted age and participant's chronological age as a measure of clock accuracy. We hypothesized that Aβ + individuals would show an increase in predicted age comparted to Aβ‐ participants. However, we found no significant differences in clock predicted age accuracy between the two groups. Both Aβ‐ and Aβ + groups had an average difference in age prediction of around −1 years (−1.03 and −0.86, respectively) and a one tailed *T*‐test revealed a *p* value of 0.86 (Figure 5). This supports the null hypothesis that biological CNS age as measured by CSF proteins is not affected in cognitively normal people with amyloid plaques.

## 4 CSF AGING PROTEIN AND PATHWAY ANALYSIS

### 4.1 Proteins predictive of increased age

Proteins with positive model weights are features that our model found to be predictive of older chronological ages. The high weights given to these proteins (relative to other proteins in the dataset) indicate that increases in their CSF concentrations were highly associated with increased age in the context of other proteins in the model. Here we explore what is known about these proteins and pathways, discuss them in the context of CSF aging and propose hypotheses based on our clock weights and correlations with age across the entire CDR = 0, Aβ‐ cohort.

#### 4.1.1 Biomarkers of neuronal damage

Our top‐ranking protein was alanine aminotransferase 1 (ALT). High levels of ALT in plasma have been associated with decreased cerebral blood flow to several areas of the brain (Chen et al., 2021). Plasma levels of ALT have also been positively associated with brain PET imaging of fludeoxyglucose, a marker to visualize neuronal injury (Nho et al., 2019). However, we hypothesize ALT may be playing a neuroprotective role in CSF. Here we observed that ALT is both significantly higher with age in CSF (Spearman = 0.50) and was the most important predictor of age in our clock overall. ALT is an enzyme that converts glutamate into alpha‐ketoglutarate and pyruvate into alanine. Molecular changes that occur during aging, such as impairment of glutamate transporters, render neurons vulnerable to excitotoxicity (Mattson & Magnus, 2006). When glutamate reuptake is inhibited, cells experience oxidative stress, neurodegeneration, and death. Glutamate excitotoxicity also plays a significant role in the pathogenesis of several acute and chronic neurological conditions including Huntington's disease and Alzheimer's disease. ALT and other glutamate degrading enzymes have been tested in‐vitro as a means to mitigate glutamate toxicity, with ALT being the most successful of these enzymes at protecting cells. (Matthews et al., 2000). Lastly, glutamate concentrations have been shown to decrease in the brain with age (Kaiser et al., 2005). Here we propose that ALT upregulation is a novel compensatory mechanism used in aging brains to decrease glutamate and mitigate age‐related vulnerability to excitotoxicity and may be an important CSF biomarker for brain health and dysfunction. Since little is known about the presence of ALT in the CNS at this time, work should be done to see if CSF levels of ALT correlate with markers of brain health and neurodegeneration and if ALT could be a therapeutic target to relieve glutamate excitotoxicity with age.

Another biomarker used in our clock to predict advancing age was neurofilament heavy polypeptide (NFH). Neurofilaments are important components of the cytoskeletal structure of neurons and are released into CSF upon axonal injury (Kušnierová et al., 2019). In addition to being highly weighted by our clock, NFH was one of the most positively correlated proteins with age (Spearman: 0.65). Recently there has been mounting interest in using neurofilaments as indicators of neurodegeneration in age‐associated neurological diseases such as AD, PD, and Amyotrophic Lateral Sclerosis (ALS) (Budelier et al., 2022; Gordon, 2020; Preische et al., 2019). We interpret higher levels of NFH with age as an increase in neurodegeneration. This fits the observation that our CSF aging model relies on markers of brain injury and dysfunction as proaging signals to predict age.

#### 4.1.2 Increased markers of neuroinflammation

Interleukin‐7 (IL‐7) is a homeostatic cytokine that is produced in a wide variety of immune cells and is required for their survival. IL‐7 has been shown to be neuro‐inflammatory, promote apoptosis of neuronal cells and activate microglia drawing them to neuronal injury sites (Carrette & Surh, 2012). We observed IL‐7 to be both positively correlated with age (Spearman = 0.22) and one of the top positively weighted proteins in our model. Inhibition of IL‐7 at spinal cord injury sites has been shown to improve recovery in mouse models (Carrette & Surh, 2012). We hypothesize IL‐7 may have potential as a therapeutic target for decreasing age‐associated neuroinflammation. Given its important role in immune cell survival, inhibition of IL‐7 in the CNS may alleviate age‐associated activation of the innate immune system and mitigate damage caused by such inflammation.

Another protein potentially involved in increased activity of the innate immune system with age is adhesion G‐protein coupled receptor G1 (GPR56). GPR56 is found to be expressed in oligodendrocyte precursor cells (OPCs), and disruption leads to a decrease in mature oligodendrocytes and OPCs resulting in hypomyelination (Ackerman et al., 2015). Given its role in myelination, it is curious that GPR56, is both positively weighted by our model as a predictor of increased age and positively correlated with age (Spearman = 0.42). One observation that might explain this is that GPR56 is highly expressed in microglia of the CNS (Ganesh et al., 2020). Thus, it may be that increased GPR56 in the CSF is another indicator of an increasingly active innate immune system in the aging brain.

#### 4.1.3 Compensatory and protective proteins

Antileukoproteinase (SLPI) is a secreted protein that inhibits leukocyte serine proteases. It is released from epithelial surfaces to protect them from attack by endogenous proteolytic enzymes (Doumas et al., 2005). Although, SLPI is expressed in mucosal linings there is increasing evidence that it plays a role in mediation of inflammation in the nervous system as well. In one study, SLPI was found to localize to neurons and astrocytes in ischemic tissue in rats following occlusion of the middle cerebral artery. In the same model, induced SLPI expression via adenovirus significantly reduced ischemic lesion size compared to controls, suggesting that SLPI may have neuroprotective properties (Wang et al., 2003). SLPI also has anti‐inflammatory functions on macrophages by suppressing their ability to release proinflammatory cytokines and nitric oxide (Doumas et al., 2005). We found SLPI to be both highly positively model weighted, indicating its predictiveness of increased age, as well as significantly positively correlated with age (Spearman = 0.48). We hypothesize this increased SLPI concentration with age in CSF may be a compensatory mechanism to counteract age associated inflammation and protect tissues from proteolytic damage by endogenous enzymes as the brain ages. Inducing SLPI expression in the brain may be another therapeutic avenue to reduce the age‐associated neuroinflammation observed in this study.

#### 4.1.4 Additional proteins

Several top positively weighted model proteins do not clearly fit into a classifiable category, their roles in brain aging and the CSF are relatively unknown, or, in one case, they were found to be a strong model predictor but not significantly correlated with age.

Pulmonary surfactant‐associated protein D (SP‐D) was the 7th highest ranked positively weighed protein in our model, but did not significantly correlate with age (*p* = 0.06). We hypothesize that proteins with such a disparity provide valuable information in predicting the age of individuals in the model, but only under specific circumstances and in conjunction with other model proteins. For example, SP‐D may become highly correlated with age when a participant has a specific disease or disorder, but is otherwise unhelpful when predicting age. Alternatively, proteins with high model predictability, but low correlations, may interact with other proteins such that the relationships can only be detected by a higher structure model. High levels of SP‐D are associated with hydrocephalus, enlargement of inner CSF spaces (Schob et al., 2016), and dementia (Nybo et al., 2007). While the literature clearly demonstrates SP‐D is connected with age‐associated conditions, understanding the circumstances in which our model uses SP‐D as a predictor of age remains a challenge.

Finally, we found four proteins on our list to be both strongly positively weighted in our model and significantly correlated with age (*p* < 0.0001); however, little else is known about their relationship with aging or their function in the central nervous system. Given the nature of other, better studied, proteins on this list, high priority should be placed on understanding the biology of these proteins in this context. These proteins include: left–right determination factor 2 (Lefty‐A, Spearman = 0.55), succinate dehydrogenase assembly factor 2, mitochondrial (SDHF2, Spearman = 0.48), prostasin (Spearman = 0.3), and syntenin‐2 (SDCB2, Spearman = 0.27).

### 4.2 Proteins predictive of decreased age

Proteins with negative weights are those that our model found to be predictive of younger chronological ages. We hypothesize that these are proteins that are lost with age and may be protective or beneficial if upregulated in the CNS. Here we discuss what is known about several of the top negatively weighted proteins found by the model and propose hypotheses based on our clock weights and correlations with age across the entire CDR = 0, Aβ‐ cohort. Unlike the previously discussed positively weighted proteins, only four out of the top 10 negatively weighted proteins are significantly negatively correlated with age, with the remaining six showing no significant trend across the cohort. Why this trend is observed so much more in the negatively weighted proteins is unknown; however, when analyzing such data, it is important to note that spearman correlations only capture monotonic increases or decreases, and that trends such as waves or peaks that may be of significant biological interest and predictive value are not captured by simple correlations.

#### 4.2.1 Loss of anti‐inflammatory and metabolism regulating proteins

Our top negatively weighted protein in the model was complement C1q tumor necrosis factor‐related protein 3 (C1QT3). C1QT3 levels in CSF were also significantly negatively correlated with chronological age (Spearman = −0.24). C1QT3 is a secreted protein expressed in many tissues and is known to have a wide range of effects including anti‐inflammatory activity, promotion of cellular differentiation and growth (Li et al., 2017), and is a known negative regulator of gluconeogenesis (Peterson et al., 2013), a process whose reduction has been shown to enhance lifespan (Hachinohe et al., 2013). Overexpression of C1QT3 in mice was also shown to improve insulin sensitivity (Peterson et al., 2013) and administration of C1QT3 in a different mouse model was shown to reduce damage following heart attacks (Yuasa et al., 2016). Work should be done to understand the effects of C1QT3 on lifespan and its role in the CNS.

#### 4.2.2 Loss of post‐transcriptional modification proteins

We found geranylgeranyl pyrophosphate synthase (GGPPS) to be both negatively weighted in our model and significantly negatively correlated with aging (Spearman = −0.24). GGPPS is a key regulator for protein prenylation, an important post translational protein modification required for cell survival, proliferation, differentiation, and migration (Palsuledesai & Distefano, 2015). One such form of prenylation is when geranylgeranyl pyrophosphate (GGPP) is attached to the cysteine at the c‐terminus of a protein. GGPP is generated from farnesyl pyrophosphate (FPP) via a chemical reaction catalyzed by GGPPS (Wang & Casey, 2016). Prenylation necessary for proper development of the cerebellum and deletion of GGPPS in neuronal progenitor cells in mice caused depletion of granule cell progenitors leading to cerebellar hypoplasia (Cheng et al., 2023). Inhibition of mevalonate production, a precursor for farnesyl and geranylgeranyl, via an inhibitor causes apoptosis and cell death in neurons and this effect can be rescued by addition of exogenous GGPP preventing cell death (Tanaka et al., 2000). Deficiency in geranylgeranyltranfersase‐1 (GGT), the protein that transfers geranylgeranyl groups from GGPP to target proteins, has been shown to reduce long‐term potentiation in the hippocampus and decrease dendritic spine density in cortical neurons in mice (Hottman et al., 2018). Thus, maintenance of GGPPS may be important to maintain GGPP levels during aging and loss of this protein may lead to neuronal vulnerability, and deficiency in memory formation through the lack of precursors needed for prenylation.

#### 4.2.3 Proteins of unknown function that negatively correlate with age

Two proteins that were in the top negatively weighted proteins had limited information regarding their functions in the CNS. These include transmembrane protein 87B (TM87B, Spearman = −0.29) and chymotrypsinogen B2 (CTRB2, Spearman = −0.16). While TM87B significantly correlated with age, CTRB2 just barely missed the cut at *p* = 0.0006. Very little is known about the normal function of either of these proteins. Given their strong weights in the model and negative associations with chronological age in the CSF, more work should be done to understand the nature of these proteins and how they affect the aging CNS.

#### 4.2.4 Negatively weighted proteins that do not correlate with age

Of the top 10 proteins negatively weighted in our model, six were not significantly correlated with age via Spearman correlation (*p* > 0.0001). These include: Glutamate receptor ionotropic, kainate 2 (GRIK2, *p* = 0.109), PAI‐2 (*p* = 0.045), platelet‐derived growth factor receptor alpha (PDGFRA, *p* = 0.002), collagen type III (*p* = 0.531), annexin A5 (*p* = 0.534), and phosphatidylcholine‐sterol acyltransferase (LCAT, *p* = 0.009). Although many of these proteins have been studied, it remains unclear how our model uses them to predict age. We hypothesize these proteins are informative only under specific circumstances or in concert with other proteins. Alternatively, these proteins could be false‐positives specific to this cohort. Here we discuss what is known about several of these proteins and how they may relate to aging.

One protein with prior aging implications is PAI‐2. Under normal conditions PAI‐2 is expressed at low levels; however, it accumulates rapidly in response to inflammatory signals around severely damaged brain tissue (Dietzmann et al., 2000). In a mouse study of cortical brain injury, PAI‐2 wild‐type mice showed significantly increased brain swelling postinjury compared to PAI‐2 knockout mice, indicating PAI‐2 levels may be a risk factor for brain edema (Griemert et al., 2019). PAI‐2 does not correlate with aging in our data but is highly negatively weighted. We observed that individuals over 85 years old did not show high levels of PAI‐2. Thus, the model may be inferring that people with high PAI‐2 levels must be under 85 years old. One hypothesis for this is a survivorship bias. It may be that high levels of PAI‐2 are a response to inflammation or brain injury and may be serving as a biomarker. Further investigation should be performed to see if individuals with high PAI‐2 in CSF are at increased risk of death as they age.

Another protein, PDGFRA, forms homodimers with itself and heterodimers with PDGFRB (Platelet‐derived growth factor receptor beta) to receive various forms of the mitogen PDGF (Platelet‐derived growth factor) (Litwack, 2018). PDGF‐BB has been demonstrated to stimulate neural precursor cell (NPC) proliferation, and PDGF‐AA has been shown to be involved in glial cell differentiation (Sil et al., 2018). PDGFR‐α‐mediated signaling is required for astrocyte and pericyte migration and maintenance of cerebral microvasculature structures (Itoh et al., 2011) and PDGF‐BB and PDGF‐CC have been shown to have neuroprotective effects across a range of diseases (Sil et al., 2018). Thus, loss of PDGFRA with age may decrease sensitivity to these neuroprotective ligands.

Collagen type III was another interesting negatively weighted model protein. Collagens are extracellular matrix proteins well known to decline with age (Varani et al., 2006). What makes collagen type III so intriguing is how important it became to our 109‐protein minimal model, rising to the most aging informative protein overall (Figure 4c). Future experiments are planned to understand why this protein becomes so informative and what other proteins in the model it is interacting with.

### 4.3 Pathways enriched in proteins used to predict aging

We sought to understand how analyzing pathways significantly enriched for proteins in our model could provide insight into the relationships between these proteins and the underlying biology providing their predictive value for aging. Here we explore several of these enriched pathways and propose hypotheses based on domain knowledge, literature, and our observations.

#### 4.3.1 Glycoprotein hormones

Glycoprotein hormones were the category most highly enriched for model weighted proteins. When examining proteins from our model enriched in the glycoprotein hormone pathway, our data shows that inhibins (INHBA, INHBB, and INHBC) are both positively weighted and significantly increase in concentration in CSF with age. Inhibins are members of the transforming growth factor‐β (TGFβ) superfamily produced by the anterior pituitary gland that block synthesis and release of Follicle‐stimulating hormone (FSH, follitropin). When FSH is released into the bloodstream it stimulates spermatogenesis in males and development of ovarian follicles in females (Santi et al., 2020). Interestingly, FSH levels tend to increase with age in plasma in both men (Araujo & Wittert, 2011) and women (Grisendi et al., 2014) counter to what one might expect given increased production of inhibin proteins. One hypothesis may be that inhibins are acting as a compensatory mechanism to help slow FSH secretion and production.

#### 4.3.2 The complement system

The complement system was another highly enriched pathway used by our model. The complement system is a highly regulated component of the innate immune system in the CNS and also plays a role in synaptic pruning of synapses by microglia in the adult hippocampus. Dysregulation of this system results in increased neuroinflammation, neurodegeneration, and cognitive impairment (Fatoba et al., 2022). Aberrant overexpression of complement components by microglia has also been linked to Huntington's disease (HD) (Singhrao et al., 1999) and shown to play a role in neurodegeneration and AD in mouse models (Hong et al., 2016). Plasma levels of components C3 and C4 have also been positively associated with metabolic disease and negatively associated with longevity in centenarians (Fu et al., 2018, 2020).

C4a is both highly positively weighted by our model and positively correlated with age. C4a is a protein released by C4 upon activation of the complement system's classical and mannose‐binding lectin pathways. These pathways lead to upregulation of C3 and activation of the innate immune system. C4a was ranked the 25th highest weighted protein in our model by absolute value, indicating its importance in predicting aging in the CSF. This provides evidence that the innate immune system is upregulated as the brain ages. Supporting this observation, C7 and C9, components of the membrane attack complex, a downstream assembly of complement system activation that lyses cells, are also both positively weighted by our model and positively correlated with age in CSF.

CFHR2 was another protein of the complement system positively weighted by our model. Although CFHR2 did not meet the significance threshold of *p* < 0.0001 to state it definitively correlates with age, it came very close (Spearman = 0.17, *p* = 0.0003). CFHR2 competes with CFH‐C3b interaction allowing activation of the complement system to proceed unhindered (Goicoechea de Jorge et al., 2013). Interestingly CFH was also found to be a positive predictor of age by our model, although to a lesser extent than CFHR2, and also correlated with increased aging. This may indicate that both activating and compensatory negatively regulating elements of the complement system are upregulated during aging.

Complement factor D (CFD) was also found to be positively weighted by our model and highly correlated with age (Spearman = 0.5). CFD is the rate limiting enzyme of C3 convertase, the enzyme which cleaves C3 into C3a, a chemoattraction molecule that increases inflammation, and C3b the molecule that tags cells for the membrane attack complex cell lysis (Barratt & Weitz, 2021).

Given the negative effects of complement system activation and its inverse correlation with mortality, we believe that inhibition of C4, a highly upstream component of two complement system branches, should be explored as a target for age‐related brain pathologies. Increased expression of CFH may also have benefits. Overall, these observations support the idea that our model uses activation of the complement system as a predictor of age, and that upregulation of this system in the CSF is positively associated with aging.

#### 4.3.3 Scavenging by class A receptors

Class A scavenger receptors are membrane bound glycoproteins involved in numerous biological functions including recognizing targets in the innate immune system, scavenging lipids in macrophages, and binding free extracellular ligands to initiate clearance. One such ligand is ferritin, which is present in the cytosol of most cells to store iron, and is made up of a heavy chain (ferritin heavy chain, FTH) and light chain (ferritin light chain, FTL) (Yu et al., 2020). Both FTH and FTL were positively weighted by our model and significantly positively correlated with aging. Notably, FTL was ranked 41st by absolute value in predictive importance by our model. It is hypothesized that extracellular ferritin is a leakage product arising from damaged cells (Kell & Pretorius, 2014) and elevated ferritin in CSF has been associated with proinflammatory neurological diseases (Zandman‐Goddard et al., 1986). This supports our observation that our clock detects increased inflammation and cellular damage products as proaging signals.

#### 4.3.4 Nonintegrin membrane‐ECM interactions

This pathway covers protein interactions between nonintegrin membrane bound extracellular matrix proteins and their ligands. Nearly all proteins in this pathway used by our model are negatively weighted indicating higher concentrations are predictive of younger ages. However, there are several positively weighted exceptions including: Transforming growth factor beta‐1 (TGF‐b1), collagen alpha‐1(X) chain (COAA1), platelet‐derived growth factor subunit A (PDGF‐AA), and tenascin. Of these, only TGF‐b1 and Tenascin are significantly correlated with age (both positive). TGF‐b1 is an anti‐inflammatory cytokine (Piras et al., 2012), and PDGF‐AA has been shown to have neuroprotective effects (Zheng et al., 2010). Tenascin in the brain becomes highly upregulated in response to neuronal injury (Chelluboina et al., 2022). Thus, we observe many of these positively weighted proteins are induced in response to neuronal stress or insult.

Of particular interest in the negatively weighted model proteins is the large representation of collagens. These include Collagen Type III, Collagen Type II, Collagen alpha‐1(I) chain: C‐term propeptide, and Collagen alpha‐2(XI) chain (COL11A2). Of these, Collagen Type III is particularly notable in that it is both one of the highest ranking negatively weighted proteins (Table 1) in the full model, and the highest ranking protein in our 109 protein minimal model, but is not significantly correlated with age. We propose that Collagen Type III and similar proteins may provide nonlinear information on age in conjunction with other proteins or are highly predictive under specific biological circumstances. Further analysis and techniques will need to be developed to understand how these non‐age‐correlative proteins are providing predictive information. Although our model highlights their importance, it remains unknown what the loss of these collagens in CSF can tell us biologically about the aging brain environment.

#### 4.3.5 TP53 regulates transcription of death receptors and ligands

The TP53 (p53) nuclear protein is the central mediator between cellular death and survival and governs many biological responses to stress including apoptosis and senescence (Kastenhuber & Lowe, 2017). One way p53 accomplishes this is upregulating TRAIL receptors that bind the TRAIL ligand to induce cell death (Willms et al., 2019). In our model, TRAIL R4 and tumor necrosis factor receptor superfamily member 10B (TRAIL R2) were positively correlated with age and positively weighted. Binding to TRAIL R2 by TRAIL induces apoptosis in tumor cells and activates pro‐inflammatory pathways, however, binding to TRAIL R4 appears to have the opposite effect and protects against TRAIL R2‐mediated cell death (Degli‐Esposti et al., 1997). This dichotomy is curious as our data shows both receptors are upregulated with age. One possible hypothesis is that cells are priming themselves for either fate in response to stress.

Another tumor necrosis factor that is both positively weighted in our model and positively correlated with age was tumor necrosis factor receptor superfamily member 6 (Fas). Fas is another cell death surface receptor that triggers apoptotic cell death via activation of caspase (Waring & Müllbacher, 1999). This further supports the hypothesis that the aging CSF is experiencing stressful conditions and priming cells for apoptotic events.

#### 4.3.6 Mitochondrial fatty acid beta‐oxidation of unsaturated fatty acids

While this pathway was enriched for model proteins, we do not have a coherent explanation for it at this time. This is due to the large representation of non‐aging correlative proteins in this pathway making interpretation of the impact on aging unfeasible baring development of more advanced analytical techniques.

## 5 DISCUSSION

In this article, we present a novel machine learning‐based CSF proteomics clock whose predictions had a 0.79 correlation with chronological age and MAE of 4.30 years in our validation cohort (ADNI) (Figure 2). Further we showed the model was interpretable and able to provide biological insight into CSF and brain aging. To do so we performed protein feature extraction on our model and analyzed both top hits and enriched pathways. We hypothesized that proteins weighted positively by our model would be frequently associated with age‐related dysfunction and disease and that proteins with negative model weights would generally be neuroprotective and enhance cell growth, and progenitor cell and tissue maintenance.

We found that top positively weighted proteins fell into two main categories: Markers of neuronal damage and signals of neuroinflammation and disease. Analysis of pathways enriched for proteins in our model supported this view with proteins of the complement system being both highly correlated with age and positively weighted, indicating an increase in innate immune system activity. Upregulation of proteins such as FTH, typically only found extracellularly upon cellular lysis, further support the observation of ongoing cellular damage with age. Additionally, upregulation of TRAIL receptors was observed with age, possibly indicating priming for proapoptotic events. An exception to this trend was SLPI, a positively weighted anti‐inflammatory, neuroprotective protein which localizes to sites of neuronal damage. We interpret this as a compensatory mechanism to combat age‐associated inflammation. Another potential novel compensatory mechanism we found was upregulation of ALT, which may be a way for the aging brain to combat glutamate excitotoxicity. We also identified several novel proteins previously unknown to be associated with CSF aging. These include Lefty‐A, SDHF2, prostasin, and SDCB2 and warrant further investigation.

We observed that top negatively weighted proteins, predicted to associate with more youthful states, were generally of a beneficial nature. Several proteins in this category had anti‐inflammatory and neuronal protective properties such as C1QT3 and PDGFRA. We also found proteins such as GGPPS, vital for post translational modifications, whose loss may lead to neuronal vulnerability, and deficient memory formation. Other proteins such as collagen type III, were of high importance to both the full and minimal models but lacked significant correlation with age. We hypothesize such proteins provide nonlinear information or become informative only in conjunction with other model proteins. Additionally, we identified two novel proteins, TM87B and CTRB2, both highly negatively weighted in our model, with TM87B negatively correlated with age and CTRB2 nearly meeting the significance threshold. Little is known about the biological functions of either protein and follow‐up studies should be done to understand their role in the aging CNS.

Next, we sought to test the utility of our model in assessing if people with age‐associated diseases, such as presymptomatic AD, experienced accelerated biological aging in CSF. This also served as an additional group on which to test our clock. We found our clock showed no significant differences in accuracy between people with presymptomatic AD versus the healthy control group. While it is well known that AD risk increases with age, this observation suggests that changes taking place in the earliest stages of Alzheimer's disease do not accelerate aging in the CNS.

Lastly, we presented a minimal version of our CSF aging clock that uses just 109 proteins to predict age, along with a novel method to generate such models we call iterative reweighting. Our 109‐protein clock performed nearly as well as the full 1157 protein clock on our ADRC test data, with a Pearson correlation with chronological age of 0.84 versus the original clock's 0.85. The clock also performed well on the ADNI validation subcohort that included only cognitively normal, amyloid negative individuals (Pearson 0.83) (Figure 4d), however, it suffered more performance loss than the full clock when predicting the entire ADNI data set which included CDR >0 and amyloid positive individuals (Pearson 0.75) (Figure 4c). While the assumption might be made that a smaller protein clock with similar performance would be preferable to the full protein clock, this may not be the case, as it is possible that such condensed clocks may not capture the full biological impacts of aging, and may be less sensitive to environmental, experimental, and disease state perturbations. Further experiments will need to be done to test the robustness of each clock under such circumstances.

## 6 CONCLUSIONS

In conclusion, this report presents a new CSF proteomics‐based clock which may predict biological aging and provides novel insights into the biology of aging in the CNS. These findings and tools may point to potential therapeutic targets for intervention in aging‐ and age‐related diseases. Future research is needed to distinguish between proteins causative of negative aging phenotypes versus proteins that are compensatory in nature. Therefore, target analysis, experimentation, and validation are needed for these potential candidate targets of aging interventions.

## AUTHOR CONTRIBUTIONS

J.M. conceived of the idea, preformed the machine learning and analysis, prepared figures, and wrote the manuscript. R.J.B. provided critical support and oversight. Y.J.S. and C.C. generated the proteomics data. M.O., A.Y., S.S., C.C, and R.J.B. provided critical feedback and helped shape the research. All authors contributed to editing and finalization of the manuscript.

## CONFLICT OF INTEREST STATEMENT

The authors have no conflicts of interest to disclose.

## Supporting information


Appendix S1.

Appendix S2.

## Data Availability

The data that support the findings of this study are available on request from the corresponding author upon approval from the Knight Alzheimer Disease Research Center (https://knightadrc.wustl.edu/) and the Alzheimer's Disease Neuroimaging Initiative (ADNI). The data are not publicly available due to privacy or ethical restrictions. All trained models and software to run the aging clocks presented in this paper on custom data sets are available upon request from the corresponding author. Code to run the full model and 109 protein minimal model is also available at https://github.com/wusm‐neurology‐batmanlab/CSF‐Proteomics‐Aging‐Clocks.
