# Correction to “An interpretable machine learning‐based cerebrospinal fluid proteomics clock for predicting age reveals novel insights into brain aging”

**DOI:** 10.1111/acel.14363

**Published:** 2024-10-08

Melendez, J., Sung, Y.J., Orr, M., Yoo, A., Schindler, S., Cruchaga, C., Bateman, R. *Aging Cell*, 2024. https://doi.org/10.1111/acel.14230

In the published version of Melendez et al. (2024), an incorrect version of Table 1 was shown.

The correct table is shown below.

**Table:** 

Protein full name	Protein	Gene	Age correlation	Model weight
**Top 10 positively weighted model proteins**				
Alanine aminotransferase 1	ALT	GPT	0.50	2.89217
Left–right determination factor 2	Lefty‐A	LEFTY2	0.55	2.624642
Succinate dehydrogenase assembly factor 2, mitochondrial	SDHF2	SDHAF2	0.48	2.597621
Neurofilament heavy polypeptide	NFH	NEFH	0.64	2.474786
Interleukin‐7	IL‐7	IL7	0.22	2.166899
Antileukoproteinase	SLPI	SLPI	0.48	2.106511
Pulmonary surfactant‐associated protein D	SP‐D	SFTPD	NS	1.997091
Adhesion G protein‐coupled receptor G1	GPR56	ADGRG1	0.42	1.925854
Prostasin	Prostasin	PRSS8	0.30	1.917393
Syntenin‐2	SDCB2	SDCBP2	0.27	1.862891
**Top 10 negatively weighted model proteins**				
Complement C1q tumor necrosis factor‐related protein 3	C1QT3	C1QTNF3	−0.24	−2.778781
Glutamate receptor ionotropic, kainate 2	GRIK2	GRIK2	NS	−2.137576
PAI‐2	PAI‐2	SERPINB2	NS	−1.823384
Platelet‐derived growth factor receptor alpha	PDGFRA	PDGFRA	NS	−1.762121
Collagen type III	Collagen Type III	COL3A1	NS	−1.717207
Transmembrane protein 87B	TM87B	TMEM87B	−0.29	−1.642012
Chymotrypsinogen B2	CTRB2	CTRB2	NS	−1.526051
Geranylgeranyl pyrophosphate synthase	GGPPS	GGPS1	−0.24	−1.41732
Annexin A5	Annexin V	ANXA5	NS	−1.403637
Phosphatidylcholine‐sterol acyltransferase	LCAT	LCAT	NS	−1.388429

We apologize for this error.

