# Supplementary material for: An interpretable machine learning‐based cerebrospinal fluid proteomics clock for predicting age reveals novel insights into brain aging
Source: Aging Cell. 2024 Jun 24;23(9):e14230. doi: 10.1111/acel.14230 (PMC11488306; doi:10.1111/acel.14230)
Supplement: Supplementary file 2 — Appendix S2. [file ACEL-23-e14230-s001.docx]

**Supplementary Materials**

Cross trained model splits:

To gauge how well our model training method performed predicting chronological age, all cognitively normal, amyloid beta negative participants were randomly split into two groups 100 times: 80% of participants were placed in a training group and 20% in a validation group. This generated 100 models on which we measured performance of each. Supplementary Figure 1 shows histograms of model performance binned by Pearson correlation (Supplementary Figure 1A) and mean estimated error (Supplementary Figure 1B). The models had a median Pearson correlation of 0.84 and median mean estimated error of 4.07.


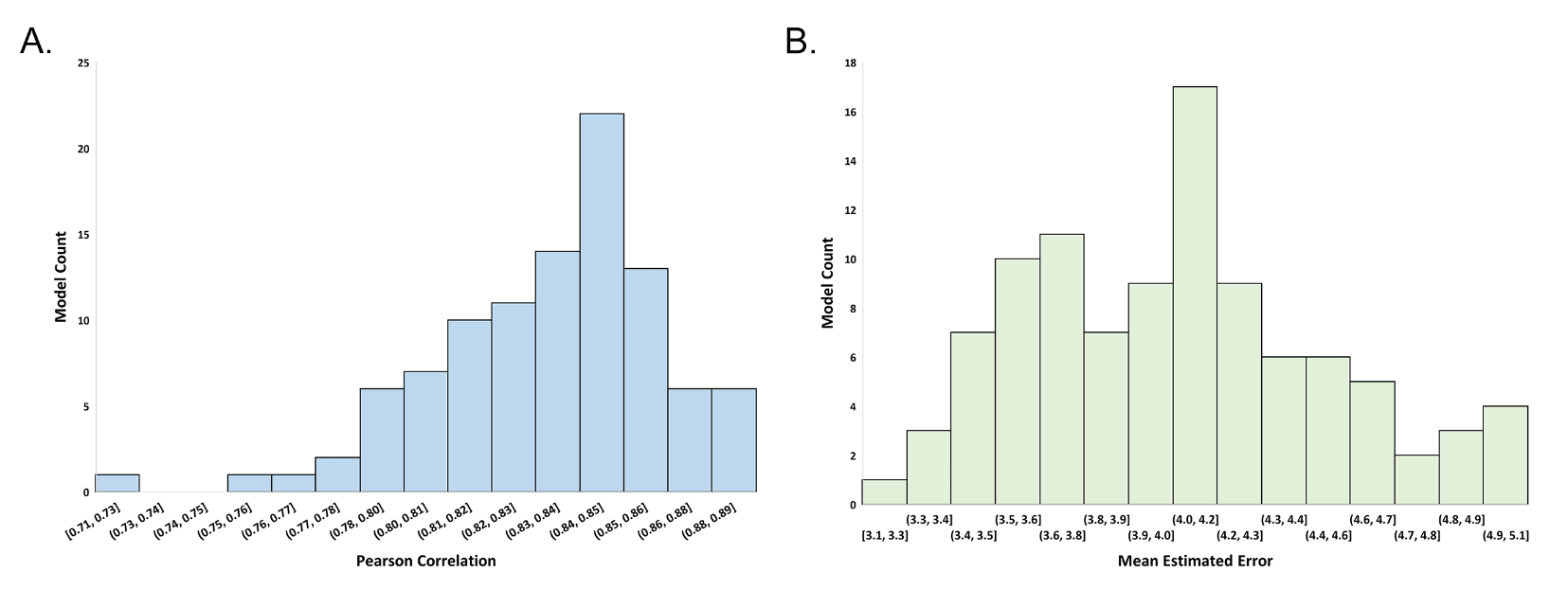


**Supplementary Figure 1: Comparison CSF aging clock training method on different splits of ADRC data.** The cognitively normal, Amyloid Beta negative ADRC CSF proteomics cohort was split 80:20 100 different ways, with 80% of the data used as training data and 20% as validation data. **(A)** A histogram of Pearson correlation for all 100 model splits. The median Pearson correlation across all models was 0.84. **(B)** A histogram of mean estimated error (MAE) for all 100 model splits. The median MAE across all model splits was 4.07.

Proteins highly enriched for in cross-trained models:

An additional way to gauge the most powerful CSF predictors of aging was to examine which proteins appeared consistently across cross-trained models. Of these, 9 proteins appeared in 100% of models (Supplementary Table 1) and 42 appeared in 90% or more of models (Supplementary files). The top 5 proteins from our main model were all present in the 100% coverage list and all proteins in the 90% plus category were present and highly ranked in the main CSF aging model. Both aging correlated and non-aging correlated proteins were present on the 100% coverage list illustrating the power of machine learning to uncover relationships that are not readily apparent from individual statistical relationships alone.

**Supplementary Table 1: Proteins predictive in 100% of cross-tested models** 9 proteins of 7,009 possible protein features were represented in all 100 cross-trained models. The top 5 ranked proteins in our main CSF aging model were all present 100% across all cross trained models.

Pathway enrichment analysis of proteins appearing in 90% of cross-trained models:

We performed pathway enrichment analysis using the 42 proteins that appeared 90% or more of the time in our cross-trained models. This was done using Metascape on the Reactome pathway knowledgebase using the same parameters used in pathway enrichment of the full model (see Methods and Materials). Using this smaller set of proteins, the only pathway we found to be enriched for was “Regulation of Complement cascade” (R-HAS-977606) (Supplementary Figure 2A). Within this pathway, C4a anaphylatoxin (C4a), Complement C4b (C4b) and Complement component C7 (C7) (Supplementary Figure 2B). It is notable that the Complement Cascade pathways were also highly enriched for by the main model (Table 2).


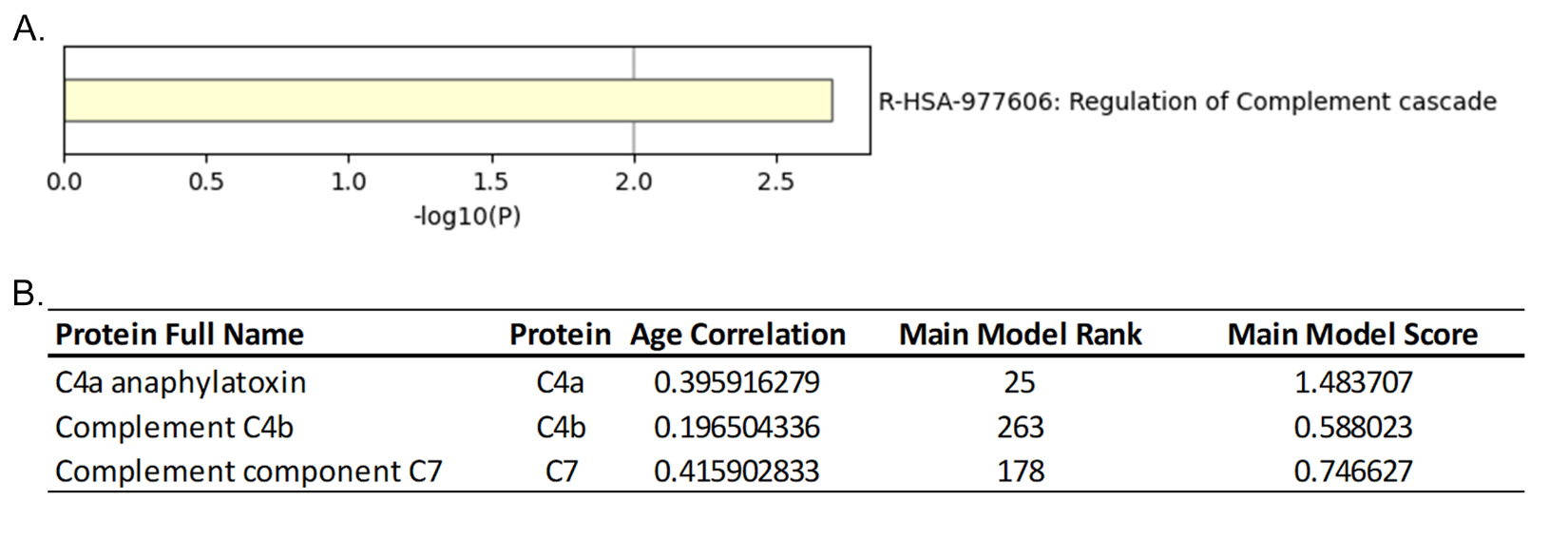


**Supplementary Figure 2: Pathway enrichment of proteins highly represented in cross-tested models**. Metascape was used to find pathways in the Reactome knowledgebase that were significantly enriched for the 42 proteins that appeared in 90% or more of cross-tested models. **(A)** Regulation of the Complement Cascade was the only pathway to be significantly enriched for in this protein set. **(B)** 3 proteins were enriched for in this pathway including C4a, C4b and C7.
